# Eye movement abnormalities in Alzheimer’s disease and other neurodegenerative dementias: insights from current evidence and priorities for future research

**DOI:** 10.3389/fopht.2025.1754941

**Published:** 2025-12-15

**Authors:** Evangelos Anagnostou, Georgios Armenis

**Affiliations:** Department of Neurology, National and Kapodistrian University of Athens, Eginition Hospital, Athens, Greece

**Keywords:** Alzheimer’s, dementia, eye movements, saccades, smooth pursuit, square-wave jerks

## Abstract

Eye movement abnormalities are increasingly recognized as early and sensitive markers of neurodegenerative dementias, particularly Alzheimer’s disease (AD). Disruptions in saccadic, antisaccadic, smooth pursuit, fixation, and naturalistic eye movement tasks reflect dysfunction in frontal, parietal, subcortical, and cerebellar circuits that are vulnerable to neurodegeneration. Studies have consistently demonstrated that AD patients show prolonged saccadic latencies, increased antisaccade error rates, reduced smooth pursuit gain, and fixation instability. Such deficits correlate with cognitive impairment, disease severity, and neuroimaging biomarkers of cortical atrophy. Comparisons with frontotemporal dementia (FTD), dementia with Lewy bodies (DLB), and posterior cortical atrophy (PCA) highlight overlapping yet distinct oculomotor profiles, suggesting diagnostic and prognostic value. Eye-tracking methodologies offer non-invasive, cost-effective tools that could complement neuropsychological and imaging assessments. However, methodological variability remains a barrier to clinical implementation. This review integrates evidence from foundational and recent studies to provide a comprehensive account of oculomotor dysfunction in AD and other dementias, emphasizing the translational potential of eye movement biomarkers in clinical practice and research.

## Introduction

Neurodegenerative dementias are characterized by progressive decline in cognition, behavior, and functional independence. Alzheimer’s disease (AD) is the most prevalent form, followed by frontotemporal dementia (FTD), dementia with Lewy bodies (DLB), and atypical syndromes such as posterior cortical atrophy (PCA). While diagnosis traditionally relies on neuropsychological testing and neuroimaging, there is growing interest in identifying sensitive, low-cost, and non-invasive biomarkers to improve early detection and differential diagnosis. Eye movements represent a promising avenue, as they rely on widespread cortical and subcortical networks that may be disrupted by dementia pathology (1–3).

Saccades, smooth pursuit, fixation, and microsaccades are fundamental to visual exploration and attention. Their neural substrates span the frontal eye fields, supplementary eye fields, dorsolateral prefrontal cortex, posterior parietal cortex, basal ganglia, superior colliculus, and cerebellum (4–6). Disruption in these systems leads to measurable oculomotor abnormalities. Importantly, these changes often precede overt clinical symptoms, making eye movement analysis an attractive candidate biomarker. This review integrates findings from various sources and synthesizes the literature on eye movement abnormalities in AD and other dementias, emphasizing the diagnostic and translational implications.

## Neural basis of eye movement control

**Figure 1:**
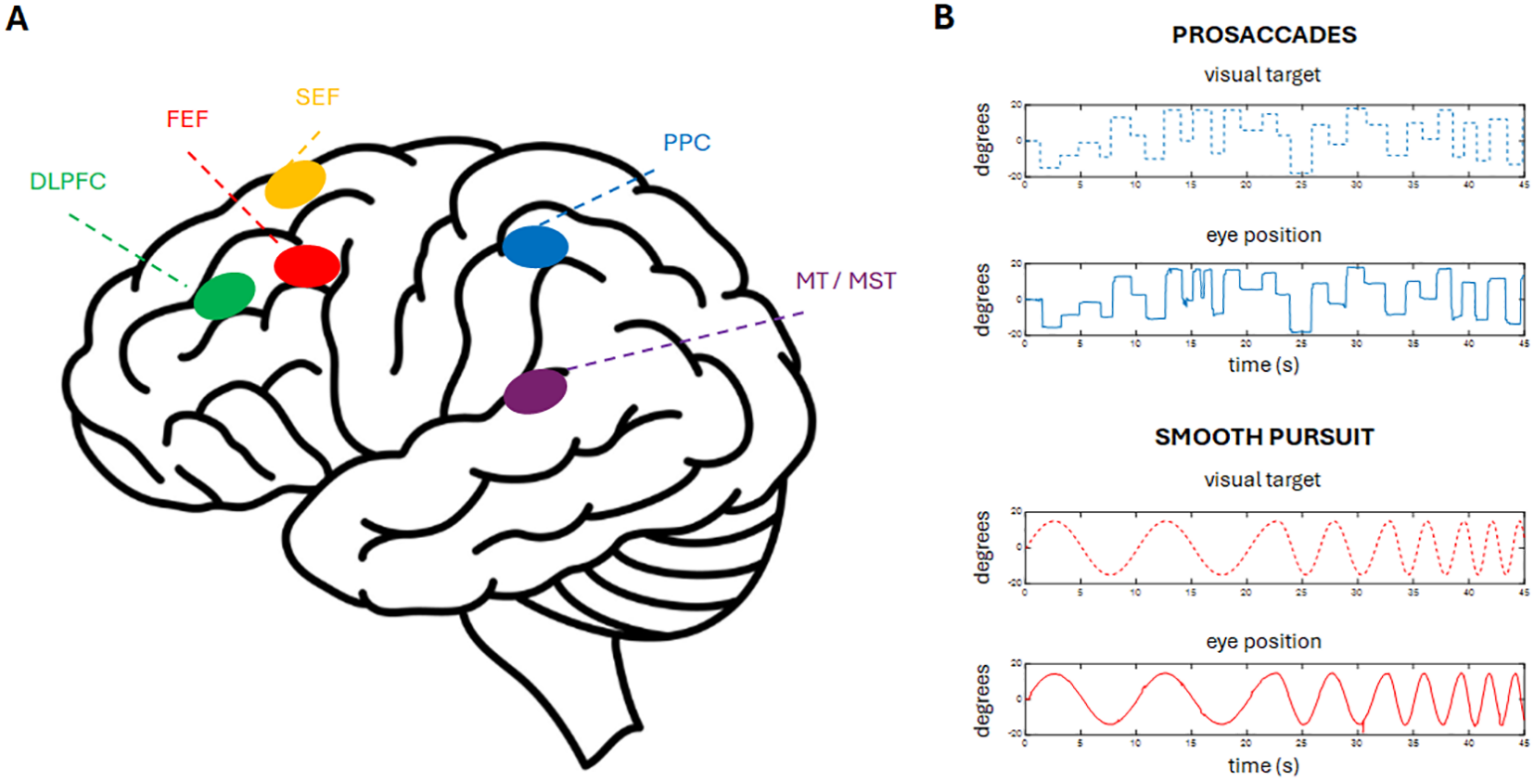
**(A)** Cortical areas involved in the generation of voluntary eye movements contain neurons that discharge during both saccades and smooth pursuit movements, with the exception of the DLPFC, which is active only during saccades, and MT/MST, which are involved exclusively in smooth pursuit. **(B)** Example of horizontal saccadic eye movements in response to a jumping visual target, and smooth pursuit eye movements in response to a sinusoidally moving visual target, recorded from a healthy 72–year–old subject. DLPFC, dorsolateral prefrontal cortex, FEF, frontal eye field, SEF, supplementary eye field, MT/MST, medial temporal/medial superior temporal area.

Eye movement control is mediated by an extensive network of cortical and subcortical regions. The frontal eye fields (FEF), the dorsolateral prefrontal cortex (DLPFC), the supplementary eye fields (SEF) (4, 5), the posterior parietal cortex and the middle temporal/medial superior temporal (MT/MST) area are the main cortical areas involved in eye movement programming (**Figure 1**). The basal ganglia regulate saccadic initiation, latency, and accuracy. The superior colliculus is central to reflexive saccade generation, and the cerebellum refines accuracy and pursuit gain (7–9).

More specifically, the generation of saccadic eye movements depends on an extensive network that spans the cerebral hemispheres, basal ganglia, cerebellum, and brainstem. Within the cerebral cortex, the FEF in the prefrontal cortex are essential for initiating voluntary saccades, while the SEF contributes to sequencing and internally guided eye movements. The DLPFC supports inhibitory control, particularly relevant for antisaccade tasks, and the posterior parietal cortex (PPC) integrates visuospatial signals for target selection (10). The basal ganglia, especially the caudate nucleus and substantia nigra pars reticulata, play a gating role: they regulate tonic inhibition of the superior colliculus and thereby influence saccadic initiation and suppression. Dysfunction here leads to abnormal saccade latencies and hypometria. The cerebellum, particularly the oculomotor vermis and fastigial nuclei, is critical for the accuracy and adaptation of saccades (11). Lesions impair the fine-tuning of amplitude, resulting in hypermetric or hypometric saccades, and contribute to coordination during smooth pursuit. At the brainstem level, the superior colliculus is the pivotal midbrain hub for reflexive saccades, integrating cortical input with sensory signals to generate rapid eye shifts. Paramedian pontine reticular formation neurons drive horizontal saccades via abducens motoneurons, while the rostral interstitial nucleus of the medial longitudinal fasciculus supports vertical saccades. Omnipause neurons in the pontine raphe maintain fixation by inhibiting burst neurons until saccade initiation (12).

By comparison, smooth pursuit relies on a distributed network spanning cortex, basal ganglia, cerebellum, and brainstem. In the cerebral hemispheres, motion processing begins in primary visual cortex and proceeds along the dorsal stream to MT/V5 and MST in lateral occipito-temporal cortex; MT extracts retinal image motion (retinal slip) while MST integrates motion with extraretinal signals (e.g., efference copy/eye velocity) and helps encode target motion in head- and world-centered coordinates. The FEF contributes to pursuit initiation, gain control, and predictive/anticipatory tracking, whereas the SEF supports sequencing, internally guided pursuit, and coordination with saccades. The PPC (including regions such as lateral intraparietal cortex (LIP)/area 7) contributes spatial attention, target selection, and the transformation of sensory motion into goal-directed signals that bias pursuit. The basal ganglia (caudate and substantia nigra pars reticulata) modulate pursuit by regulating inhibitory output to midbrain/brainstem oculomotor structures, thereby influencing initiation, maintenance, and context-dependent gain. The cerebellum is pivotal for calibration and adaptation: the flocculus/paraflocculus adjust pursuit gain and phase during continuous tracking, while the oculomotor vermis and fastigial oculomotor region shape transient dynamics and help coordinate catch-up saccades during combined tracking. In the brainstem, cortical and MST signals descend via the dorsolateral pontine nuclei and nucleus reticularis tegmenti pontis to the cerebellum; output from vestibulo-cerebellar circuits acts through vestibular nuclei and premotor pathways to extraocular motoneurons. Disruption anywhere along this chain—MT/MST (sensory), FEF/SEF/PPC (sensorimotor transformation), basal ganglia (gating), cerebellum (adaptation), or pontine/vestibular relays—yields characteristic low-gain, phase-lagged, or erratic pursuit with increased reliance on catch-up saccades.

In AD, widespread degeneration of frontal and parietal cortices, as well as cholinergic deficits (13), disrupt these systems. This may result in delayed saccade initiation, poor antisaccade performance, and reduced smooth pursuit quality. Subcortical involvement is expected to cause hypometric saccades and increased variability. The convergence of deficits across these circuits highlights the sensitivity of oculomotor measures to distributed neuropathology.

## Saccadic and antisaccadic abnormalities in Alzheimer’s disease

Since the metrics of saccades (amplitude, velocity, acceleration, and deceleration) are tightly linked to cerebellar and brainstem saccadic circuits, they are of less interest when examining patients with cortical disturbances such as those prevalent in dementia. For these patients, tasks that probe and manipulate latency (reaction time) and thus the programming of saccade initiation are more relevant. Accordingly, most researchers analyze the influence of different saccadic tasks—reflexive saccades, antisaccades, and memory-guided saccades—on saccadic latency by comparing patients with dementia to age-matched controls.

Reflexive saccades (prosaccades) are rapid, stimulus-driven eye movements toward suddenly appearing peripheral targets. In this task, the subject fixates centrally; when a target appears in the periphery, the subject looks at it. Antisaccades, by contrast, require suppression of the reflexive response and the generation of a voluntary saccade in the opposite direction (14). Here, the subject fixates centrally, and when a target appears, they must look toward the mirror-opposite location. Memory-guided saccades are designed to test spatial working memory. In this task, a target briefly flashes at a peripheral location and then disappears; after a delay, a cue instructs the subject to saccade to the remembered location (15). While the term prosaccades is often used interchangeably with visually guided or reflexive saccades, there is another subtype of prosaccades known as predictive saccades. These occur when subjects follow targets that alternate regularly between two locations at a fixed rate - an artificial situation that is unlikely to occur outside laboratory conditions. Hence, predictive saccades represent an internal model of rhythmic structure governed predominantly by the cerebellum, rather than an exogenous information elicited response (16).

Saccadic deficits are consistently reported in AD. Prosaccade tasks demonstrate increased latency (2, 17–28) and latency variability (3, 29–33), increased gap-effect (2) and high perseveration, reduced gain (32, 34, 35) or reduced accuracy (18, 19, 28, 36) and greater variability in amplitude and velocity (3, 31). Antisaccade paradigms, requiring the suppression of a reflexive saccade towards a stimulus and the generation of a voluntary movement in the opposite direction, are particularly sensitive to executive dysfunction. AD patients demonstrate longer latencies (19, 25, 30) and latency variability (30), elevated antisaccade error rates (2, 17, 19, 21, 22, 24–27, 30, 32–34, 37–42), lower latency for errors (21) and low error correction compared with controls (17–19, 21, 25–27, 33, 34, 40, 41), with prolonged correction time (29), error rates correlate with disease severity (22, 43). Increased anti–effect (i.e. the difference between pro– and antisaccade reaction times) and gap effect (30) and reduced accuracy (18, 19) have also been reported. Predictive saccade paradigms, requiring sustained endogenous eye movement production in response to predictable enviromental cues are suitable for testing motor planning and spatial working memory in executive function (44). Predictive saccades demonstrate longer latencies (25, 45) and increased latency variability (29, 32), increased variability in predictive performance (33), as well as increased gain and gain variability (29). Partial correlation analyses indicated that global cognitive performance is positively linked to pro– and anti–saccade accuracy, as well as anti–saccade error correction, while being negatively related to pro–saccade latency. Word fluency also showed a positive association with saccade accuracy and error correction (18). Collectively, these results suggest that eye–movement measures—particularly those derived from pro– and anti–saccade tasks—are closely tied to multiple cognitive domains in patients with mild to moderate AD.

### Smooth pursuit deficits

Smooth pursuit—the ability to continuously track moving stimuli—is impaired in AD. In laboratory settings, smooth pursuit is typically elicited in two ways: by presenting a sinusoidally oscillating visual target and instructing the subject to follow it, or by displaying a stimulus moving in a ramp–like trajectory. In both paradigms, the primary parameter assessed is pursuit gain, defined as the ratio of eye velocity to target velocity. Impaired smooth pursuit is characterized by reduced gain due to the replacement of smooth tracking with frequent catch–up saccades.

Most studies report that patients with AD exhibit reduced pursuit gain (1, 27, 34, 36, 46–48), although some investigations have not confirmed this finding (18, 36, 49, 50). AD patients are prone to increased pursuit error—defined as the difference between the target position and the gaze position during a pursuit task (19). They also show lower peak velocity and a reduced proportion of time spent pursuing the target (36, 51), a higher frequency of large–amplitude saccadic intrusions in the direction of target motion (47, 48, 51), increased latency (27, 34), more frequent square–wave jerks (SWJs) during pursuit (52), and prolonged start–up duration (17). Start–up duration refers to the time from the initiation of target motion until the eyes successfully track onto the target, and thus differs slightly from onset latency, which denotes the onset of any smooth eye movement during the pursuit task, regardless of whether the target is successfully foveated.

These abnormalities have been attributed to reduced metabolism in the right posterior middle temporal gyrus, a region located near the middle temporal complex—an area critical for motion processing and pursuit initiation (19). Moreover, these impairments likely reflect deficits in attentional allocation as well as general motor precision.

### Fixation instability and microsaccades

Fixation stability relies on the suppression of unwanted saccades and the precise control of microsaccades (6). In AD, fixation is typically unstable (25, 28), characterized by frequent large intrusive saccades (35, 36, 53), with this deficit progressively worsening over 9–18 months of follow–up (45). Impaired fixation in AD is also reflected in global oculomotor metrics, such as a reduced maximum fixation duration (36, 53). Reports describe an increased—or sometimes normal—frequency of SWJs (36, 46, 53). Notably, in the absence of a visual fixation target, SWJs are triggered rather than suppressed in individuals with AD, a pattern opposite to that of healthy controls (53, 54).

Microsaccades, which are physiological micromovements in healthy individuals, exhibit abnormal dynamics in AD, predominantly showing oblique trajectories rather than the strictly horizontal trajectories observed in controls (53, 55). Together, these findings highlight the value of high–resolution eye–tracking for detecting subtle oculomotor signatures of dementia.

### Comparisons with other neurodegenerative dementias

Comparative studies indicate that distinct oculomotor profiles may aid differential diagnosis. In FTD, antisaccade error rate is high, comparable to AD, reflecting profound frontal executive dysfunction. Behavioral–variant–FTD and non–fluent–Primary Progressive Aphasia patients, however, unlike AD patients, show high error correction, consistent with a relatively unaffected monitoring capacity, especially when the underlying pathology is TDP–43 (19, 25–27, 34, 56–58). Reduced prosaccade vertical amplitude and velocity is also reported in contrast with AD findings (56, 59). In DLB, slowed saccade initiation, hypometric saccades, impaired attentional disengagement, increased uncorrected antisaccade errors (executive and monitoring dysfunction) are more prominent than in AD, reflecting striatal and parietal involvement (25, 42). PCA, often underpinned by AD pathology, demonstrates marked deficits in prosaccades (inability to disengage from a target to fixate a new one, ‘sticky fixation’) and fixation stability (large intrusive saccades) consistent with parietal and occipital cortical atrophy (36, 46, 53). These distinctions underscore the potential of eye movement assessments in differential diagnosis. A summary of oculomotor phenotyping of the above diseases can be found in **Table 1**.

**Table 1:** Summary table of eye movement abnormalities in neurodegenerative dementias.

	Alzheimer’s disease	Posterior cortical atrophy	Dementia with Lewy bodies/Parkinson’s disease dementia	Frontotemporal dementia
Prosaccades	increased latency (2, 17–28) and latency variability (3, 29–33), increased gap–effect (2), high perseveration (28), increased time to fixate the target and increased number of saccades to reach the target (36), decreased gain (25, 32, 34, 35), decreased accuracy (18, 19, 28, 36), increased variability of accuracy and speed (3, 31)	decreased accuracy (~AD), increased time to fixate the target (>AD) (36, 46), increased number of saccades to reach the target (>AD) (36), increased gap/overlap effect (‘sticky fixation’) (46)	increased latency (25) and decreased gain (<AD (42)), absence of express saccades and decreased gap/overlap effect, decreased accuracy and decreased peak velocity and increased variability of all saccade parameters (62)	no–abnormalities in behavioral–variant–FTD (19) and Primary Progressive Aphasia (25, 27, 34), but subtle latency prolongation in other studies (57, 58, 60), decreased vertical amplitude (56), decreased vertical velocity (56, 59)
Antisaccades	increased latency (19, 25, 30), increased variability of latency (30) and increased errors (2, 17, 19, 21, 22, 24–27, 30, 32–34, 37–42), lower latency for errors (21), low error correction (17–19, 21, 25–27, 33, 34, 40, 41) with prolonged correction time (29), increased anti–effect and gap effect, increased anticipations (30), decreased accuracy (18, 19)	no studies available	increased latency and increased uncorrected errors (>AD) (25, 42)	increased errors but self–corrected in behavioral–variant–FTD and non–fluent Primary Progressive Aphasia, but normal in semantic dementia (25–27, 34, 56), in a comparative study behavioral–variant–FTD showed less errors than AD (19), increased errors with low correction rate in FTD–Pick (26), increased errors with unreported correction rate in FTD and Primary Progressive Aphasia (57, 58), increased latency and increased early saccades in behavioral–variant–FTD and Primary Progressive Aphasia (57, 60)
Predictive saccades	increased latency (24, 25) and latency variability (29, 32) and increased variability in predictive performance (33), increased gain and gain variability (29)	no studies available	increased latency and decreased saccade prediction (25)	increased latency in behavioral–variant–FTD (60), higher latencies in shorter predictable interstimulus intervals and less predictive saccades (61)
Memory–guided saccades	increased latency (25) and decreased accuracy (19)	no studies available	increased latency and decreased express saccades [gap paradigm] (25)	increased latency and errors in behavioral–variant–FTD (60)
Fixation stability	increased large intrusive saccades (36, 48, 53), decreased maximum fixation duration (36, 53), normal intrusive saccades (~controls) at baseline but increased over 9–18 months of follow up (>controls) (45), high impersistence (28), increased fixation instability (25)	increased large intrusive saccades (~AD (36) or >AD (46)), decreased maximum fixation duration (<AD) (36, 53)	no studies available	decreased longest period of fixation in behavioral–variant–FTD, (53, 56)
Square–wave jerks	increased (46, 53) or normal in other studies (36, 53), SWJs are facilitated rather than suppressed in the absence of visual fixation (53, 54), increased during smooth pursuit (52), microssacades with oblique direction (53.55)	no abnormalities (36, 53)	no studies available	increased number of small and normal number of large SWJs in behavioral–variant–FTD (53, 56)
Smooth pursuit	decreased (1, 27, 34, 46–48) or normal gain in other studies (18, 36, 49, 50), increased latency (27, 34), increased error (19), large–amplitude saccadic intrusions in the direction of target motion (47, 48, 51), lower values of average peak velocity of smooth pursuit and of proportion of time pursuing the target (36, 51), SWJs during pursuit (52)	normal gain, decreased proportion of time pursuing the target (36), decreased gain (~AD) (46)	no studies available	decreased gain in FTD, normal in semantic dementia and non–fluent Primary Progressive Aphasia (27, 34), increased latency in FTD, normal in semantic dementia and non–fluent Primary Progressive Aphasia (27), decreased vertical gain in behavioral–variant–FTD, normal in Primary Progressive Aphasia (19), normal visual guided and decreased predictive gain (49)

~ similar to, > more than, < less than.

## Discussion

Eye movement abnormalities provide a non–invasive, cost–effective, and sensitive window into neural dysfunction in Alzheimer’s disease and other dementias. Modern eye trackers provide a powerful means for non–invasive, high–precision assessment of oculomotor performance across a variety of clinically relevant tasks. Most stimulation paradigms are already integrated into commercial video–oculography systems, which also offer software–based extraction of key outcome metrics. Additionally, some setups support raw–data export, enabling offline analysis with custom–developed programs in individual laboratories for research purposes. Deficits in saccadic latency, antisaccade performance, pursuit gain, fixation stability, and naturalistic viewing are consistent hallmarks of AD. Comparisons with FTD, DLB, and PCA reveal overlapping but distinct profiles that may aid differential diagnosis. By contrast, instrument–derived eye–movement metrics with a long tradition in clinical neurology—such as saccadic peak velocity and nystagmus characteristics including mean slow–phase velocity and their dependence on eye–in–orbit and head–in–space position—are of limited utility in neurodegenerative dementias. This is because the infratentorial brain structures that primarily govern these metrics are not central to the pathophysiology of dementia.

Despite promising findings, methodological variability remains a major limitation in the field. Differences in task design, sample size, disease stage, and analytical approaches contribute substantially to heterogeneity across studies. High–resolution eye trackers and standardized protocols are essential for the reliable measurement of specific metrics—such as saccade peak velocity—whereas more affordable tracking devices may be sufficient for other oculomotor parameters, such as error rates in antisaccade tasks. Moreover, future studies should aim to balance controlled laboratory paradigms with more naturalistic tasks (63) to enhance clinical applicability. Perhaps the most critical issue in existing research, however, is the lack of neuropathological and/or neurochemical confirmation of AD diagnosis. The combination of reduced CSF Aβ42 (or the Aβ42/Aβ40 ratio) and elevated CSF phosphorylated tau (p–tau) currently provides the most reliable *in vivo* proxy for AD pathology, approximating neuropathological confirmation during life (64). In contrast, most available eye movement studies rely solely on neuropsychological profiles for clinical AD diagnosis – a practice long recognized as insufficient, given that syndromal AD does not consistently reflect underlying AD neuropathology (65, 66).

Methodological standardization and integration with multimodal biomarkers will be essential for clinical translation. In addition, larger longitudinal cohorts are needed to determine which oculomotor metrics are most relevant – both for refining differential diagnosis among neurodegenerative dementias and, more importantly, for serving as early, potentially preclinical biomarkers and follow–up measures. Overall, oculomotor assessments represent a promising frontier for dementia research and clinical practice.
